# Supplementary material for: Factors associated with students’ unwillingness to voluntarily donate blood in the Eastern Region of Ghana
Source: PLoS One. 2026 May 6;21(5):e0348094. doi: 10.1371/journal.pone.0348094 (PMC13148664; doi:10.1371/journal.pone.0348094)
Supplement: Attachment — Submitted filename: Reviewes_Blood donation.docx [file pone.0348094.s002.docx]

1. **Title and Conceptual Alignment**

- The title is misleading. The analysis focuses on *willingness or unwillingness to voluntarily donate blood (VBD)* rather than actual *low blood donation* or *low turnout*. These concepts represent different stages of the blood donation pathway (intention vs behaviour)..
- Authors should make explicit and clear their view on whether the primary problem is low blood donation, low turnout, non-voluntary donation, or unwillingness and should maintain consistency across the title, objecting, literature review, results, and conclusions.
- If unwillingness is conceptualised as a determinant of low blood donation, this should be explicitly stated and reflected in the title and analytical framing.

2. **Literature Review and Justification**

- The literature review and justification are conceptually inconsistent. They alternate between factors influencing willingness, turnout, and low donation as if they are interchangeable.
- The justification is therefore double-barrelled and obscures the primary research problem.
- The literature review should be reframed to focus on factors influencing low blood donation, with willingness clearly positioned as one explanatory construct if retained as the outcome.

3. **Study Design and Methods Description**

- The study includes inferential analyses; therefore, it should be described as a cross-sectional analytical study, not purely descriptive or simplt cross-sectional study.
- Clarify whether the questionnaire was self-administered or interviewer-administered.

4. **Sample Size and Sampling Procedures**

- The manuscript does not explain how the student sample size (n = 2,467) was determined, nor whether this represented a minimum required sample size. A formal sample size calculation should be described.
- The sampling procedures lack clarity. The authors should:
  - Clearly state the total number of second-cycle and tertiary schools in the Eastern Region at the time of the study.
  - Clarify whether “visited schools” represents all existing schools or only those previously reached by the blood service.
  - Justify the selection (sampling) of 21 second-cycle and 3 tertiary schools, particularly given that some districts reportedly had no schools and that only five tertiary institutions exist.
  - Ensure the word selected does not mean sampled for methodological clarity.
  - Clearly describe how students were sampled within schools (e.g., sampling frame, systematic or simple random sampling).

5. **Statistical Analysis**

- The study employed multistage sampling (districts, schools, students), yet the hierarchical structure is not adequately addressed statisitically.
- The authors should clearly state the primary unit (cluster)—district or school.
- It should be clarified whether clustering effects at district or school level were assessed, including their contribution to variance.
- Describe how the regression model accounted for clustering and hierarchy (e.g., clustered standard errors, multilevel modelling), and justify the chosen approach.
- The correct term is multivariable analysis, not multivariate analysis, as only one outcome variable was analysed.
- The manuscript should clearly explain how variables were selected for inclusion in the multivariable model (e.g., based on bivariate significance, theoretical relevance).

6. **Results Presentation**

- The Results section is incomplete. Although tables include both descriptive and multivariable findings, the text describes mainly descriptive results.
- The narrative should explicitly interpret adjusted results by stating:
  - Whether an association exists
  - The strength of association (adjusted odds ratios with 95% CIs)
  - The direction of association
- Ensure consistency in reporting proportions (e.g., x% [n/N]) throughout the Results section.

7. **Discussion**

- The Discussion is largely framed around statistical significance versus non-significance and does not adequately interpret the magnitude or direction of associations.
- The Discussion should focus on how specific factors increase or decrease willingness to donate blood.
