# Supplementary material for: Factors associated with students’ unwillingness to voluntarily donate blood in the Eastern Region of Ghana
Source: PLoS One. 2026 May 6;21(5):e0348094. doi: 10.1371/journal.pone.0348094 (PMC13148664; doi:10.1371/journal.pone.0348094)
Supplement: Attachment — Submitted filename: Response_to_Reviewers_auresp_2.docx [file pone.0348094.s005.docx]

Koforidua Technical University Department of Medical Lab. Science

P.O. Box KF 981, Koforidua,

Eastern Region, Ghana.

6^th^ April, 2026.

**The Editor,**

**PLOS One.**

Dear Sir/madam,

**RESPONSE TO REVIEWERS COMMENTS**

I write to respond to reviewers' comments on the manuscript: Factors associated with students’ unwillingness to voluntarily donate blood in the Eastern Region of Ghana. PONE-D-25-60625R1

**Response to general journal comments**

Please the reviewer comments did not include a recommendation to cite specific previously published works.

The reference list has been checked for correctness and completeness.

No retracted paper has been cited as at the time of submission of this response.

**Response to reviewer 2 comments**

The new text on sample size and response rate is appreciated. However, it is currently situated in Methods, whereas conventionally this should be in Results (the first Result reported): “A total of 2,573 students from the 24 institutions were invited to participate in the study using systematic random sampling. Out of these, 2,467 students filled the questionnaire, yielding a response rate of 95.9%. However, 267 responses were excluded based on the study exclusion criteria, resulting in a final sample of 2,200 respondents.”

**Response:** Please, the text has been moved to the result section. page 11

Relatedly, the 95.9% response rate for a voluntary survey is extraordinarily (some would say suspiciously) high. Can the authors please add a comment on this in the limitations section of the Discussion, noting whether there were any incentives (formal or otherwise), whether the authors feel this influenced participants’ responses, and if so, in what way and direction?

**Response:** Please, this has been addressed in the limitation section on page 21 and highlighted in red.

The high response rate recorded (95.9%) may be attributed to the direct administration of questionnaires to students, as no incentive was given. However, the schools assigned a coordinator who introduced the survey team to the students. This approach may have influenced students’ participation in the study. Students may have felt obliged to complete the questionnaire or provide favourable responses, despite the study being clearly explained as voluntary.

We appreciate your time and cooperation. Thank you.

Yours faithfully,

Dr Augustine Badu,

Corresponding author.
